# Influence of Eating Schedule on the Postprandial Response: Gender Differences

**DOI:** 10.3390/nu11020401

**Published:** 2019-02-14

**Authors:** Marcela Masihy, Hugo Monrroy, Giulio Borghi, Teodora Pribic, Carmen Galan, Adoracion Nieto, Anna Accarino, Fernando Azpiroz

**Affiliations:** 1 Departament de Medicina, Universitat Autònoma de Barcelona, 08193 Bellaterra (Cerdanyola del Vallès), Spain; marcelamasihy@gmail.com (M.M.); hmonrroy@gmail.com (H.M.); giulio.borghi@gmail.com (G.B.); teodora.pribic@gmail.com (T.P.); cgalanhidalgo@gmail.com (C.G.); anietoruiz7@gmail.com (A.N.); aaccarino@telefonica.net (A.A.); 2 Department of Experimental Medicine (Medical Pathophysiology, Food Science and Endocrinology Section) “Sapienza” University of Rome, 00185 Rome, Italy

**Keywords:** meal ingestion, meal schedule, eating habits, postprandial sensations, hedonic response, homeostatic responses, gender differences

## Abstract

Ingestion of a meal induces conscious sensations depending of the characteristics of the meal and the predisposition of the eater. We hypothesized that the eating schedule plays a conditioning role, specifically, that an extemporaneous meal is less rewarding than when eaten at the habitual schedule. We conducted a randomized parallel trial in 10 women and 10 men comparing the responses to a consistent savoury lunch-type meal (stewed beans) eaten at the habitual afternoon schedule or at an unconventional time in the morning. Schedule and gender differences were analyzed by repeated measures analysis of covariance. In women, the sensory experience induced by the probe meal, particularly postprandial satisfaction, was weaker when eaten at an unconventional time for breakfast. Men were resilient to the schedule effect and experienced the same sensations regardless of the timing of ingestion; the effect of the eating schedule was significantly more pronounced in women for fullness (F(1,55) = 14.9; *p* < 0.001), digestive well-being (F(1,36.8) = 22.3; *p* < 0.001), mood (F(1,12.4) = 13.8; *p* < 0.001), and anxiety (F(1,11.9) = 10.9; *p* = 0.001). No differences in the physiological responses induced by the afternoon and morning meals were detected either in women or men. Our data indicate that women are more susceptible to changes in meal schedule than men.

## 1. Introduction

The activity of the digestive system changes in response to ingestion of a meal from a stereotyped fasting pattern to a tightly controlled digestive pattern, which is part of the broader homeostatic response to the meal [1,2]. Ingestion also induces sensations, both homeostatic (satiation, fullness) and hedonic (meal wanting, liking, digestive well-being, changes in mood), depending on the characteristics of the meal [1]. The responses also depend on the predisposition of the eater, which can be modulated by a series of conditioning factors, such as previous meals, e.g., breakfast on the study day [3], how palatable the meal is found to be [4], and cognitive factors [5]. In this context, we hypothesized that the eating time also plays a conditioning role, specifically, that an extemporaneous meal is less enjoyable than when eaten within the habitual schedule. The effect of meal schedule on the postprandial experience may be highly relevant due to its potential influence on eating habits, energy balance, and the control of body weight [6,7,8].

We have recently shown that women enjoy and tolerate smaller meals more than men [9]. Based on these data, we further postulated that this susceptibility also involves the schedule effect. Hence, we designed a study to compare the schedule effect between women and men. In each group, the schedule effect was measured by comparing the responses to a consistent savoury lunch (stewed beans) eaten at the habitual lunch-time for the local population (in the early afternoon) or at an unconventional time (in the morning). As in previous studies, the specific contribution of sex, i.e., biology, versus gender, i.e., roles, in the responses to meal ingestion was not investigated, and the term gender is used in a broad sense to encompass the differences between women and men [9]. 

## 2. Material and Methods

### 2.1. Participants

Twenty healthy non-obese, non-dieting, and weight-stable subjects (10 women, 10 men) without a history of gastrointestinal symptoms were recruited by public advertising to participate in the study. Exclusion criteria were chronic health conditions, prior obesity, use of medications (except occasional use of nonsteroidal anti-inflammatory drugs and antihistamines), a history of anosmia and ageusia, current dieting or any pattern of selective eating, such as vegetarianism, alcohol abuse, and use of recreational drugs. Absence of current digestive symptoms was verified using a standard abdominal symptom questionnaire (no symptom >2 on a 0–10 scale). Psychological and eating disorders were excluded using the following tests: the Hospital Anxiety and Depression scale (HAD), the Dutch Eating Behaviour Questionnaire (DEBQ: Emotional eating, External eating, Restrained eating), and the Physical Anhedonia Scale (PAS). Candidates were asked whether they liked a list of foodstuffs and those who did not like the probe meal to be used (see below) were not included. In order to reduce variations in gut function related to the menstrual cycle, women were studied during the follicular phase of the menstrual cycle (days 5–15).

The research was conducted according to the Declaration of Helsinki. The protocol for the study had been previously approved by the Institutional Review Board of the University Hospital Vall d’Hebron, and all participants gave written informed consent.

### 2.2. Experimental Design

Gender differences in the effect of meal schedule were studied in women and men in a parallel, open-label, single-centre trial performed between August 2018 and November 2018. The study protocol was registered with the ClinicalTrials.gov NCT03773432. All co-authors had access to the study data and reviewed and approved the final manuscript.

### 2.3. General Procedure

Each participant underwent two studies in random order on separate days within a 1-week interval. One day, participants ingested the probe meal (see below) in the afternoon, i.e., on the habitual schedule, and the other day in the morning, i.e., an unconventional schedule. Participants were blind to the specific aim and primary outcome of the study; they were told that the study investigated the responses to a meal under different conditions without specifying the schedule effect. For both study days, participants were instructed to refrain from strenuous physical activity and to refrain from smoking on the study day. Participants were instructed to eat a standard dinner at 10 p.m. the day before (100 g grilled chicken, 50 g rice, 50 g white bread, and one apple; 503 Kcal, 7 g lipids, 82 g carbohydrates, 30 g protein; glycemic index 47.1). The day of the afternoon test, they were instructed to eat a standard breakfast (tea or coffee, regular or decaffeinated depending on their habits, with semi-skimmed milk and biscuits; 181 Kcal, 5 g lipids, 27 g carbohydrate, 7 g protein) at 9 a.m. and to report to the laboratory, where the probe meal was administered 5 h after breakfast. The day of the morning test, the probe meal was administered at 9 a.m. after an overnight fast. Studies were conducted with participants sitting alone with one investigator in a quiet, isolated room.

### 2.4. Probe Meal

The probe meal consisted of 300 g stewed beans (withe beans with pork sausage and bacon; Fabada Asturiana, Litoral, Barcelona, España), 35 g bread (Panecillo Redondo Rustico, Hacendado, Valencia, España) and 100 mL water; glycemic index 28.5 (Table 1). Based on preliminary studies, participants were instructed to consume the meal in 12 minutes at a comfortable eating rate.

### 2.5. Main Outcomes

Perception of homeostatic and hedonic sensations was measured as follows. Five 10-cm scales graded from −5 to +5 were used to measure: (a) meal wanting (impossible/eager), (b) meal liking (very disagreeable/very agreeable), (c) hunger/satiety (extremely hungry/completely satiated), (d) digestive well-being (extremely unpleasant sensation/extremely pleasant sensation), and (e) mood (negative/positive); a 10-cm scale graded from 0 (not at all) to 10 (very much) was used to measure abdominal bloating–fullness. Subjects received standard instructions on how to fill out scales (9). The wanting scale was only scored at the presentation of a meal. The liking scale was only scored at the end of ingestion. The rest of the scales were scored at 5-minute intervals 10 minutes before and 20 minutes after ingestion, and at 10-minute intervals up to 60 minutes after the probe meal.

### 2.6. Additional Measures

Other sensations. Three additional scales were used to measure somnolence (−5, very alert/+5, very sleepy), anxiety (−5, very relaxed/+5, very anxious), and abdominal discomfort–pain (0, not at all/10, very intense) before and after the meal as described above.

Blood pressure was measured (using an M6AC, Omron, Kyoto, Japan) during baseline, the early postprandial period, and 30 minutes and 60 minutes after ingestion.

Assessment of Heart Rate Variability. Continuous cardiac interbeat intervals (IBI) were recorded using a lightweight device (Bittium Faros 360°, Mega Electronics, Kuopio, Finland) via five surface electrodes at a 500 Hz sampling rate. Heart rate variability (HRV) was assessed over 5-minute recording periods at baseline, the early postprandial period, and 30 minutes and 60 minutes after ingestion. HRV analysis of the exported data was performed using dedicated HRV software (Kubios Premium ver. 3.1.0) as previously described [10]. Prior to HRV computation, all IBI data were visually inspected for correctness and then underwent automatic artifact correction. HRV spectra were calculated by an autoregressive transformation. High-frequency power data (0.15–0.40 Hz) were computed as normalized units [11,12]. Respiratory rate was calculated using an ECG-derived respiration software within the HRV analysis package, as previously described [13].

Antral and gallbladder imaging. Ultrasound images of the gastric antrum and the gallbladder were obtained using a Chison ultrasound scanner (ECO1; Chison, Jiangsu, China) with an abdominal 3.5-Hz probe (C3A; Chison, Jiangsu, China), as previously described in detail [14,15,16,17]. In brief, images were obtained with the subjects seated and leaning slightly backwards in a chair. Gastric images between antral contractions were obtained in triplicate before and at 5 minutes, 30 minutes, and 60 minutes after the meal; using the superior mesenteric vein and the aorta as landmarks, the outer profile and the cross-sectional area of the antrum were measured using the built-in calliper and measurement tool. Longitudinal and transverse gallbladder images were obtained before and 60 minutes after the meal to measure the three maximal axes and the gallbladder volume by the ellipsoid method.

### 2.7. Statistical Analysis

Statistical analysis was performed using the Stata Software for Windows, (StataCorp. 2017. Stata Statistical Software: Release 15. College Station, TX: StataCorp LLC).

Based on previous data on the differences in postprandial digestive well-being between women and men [1], the sample size was estimated to detect a 10% gender difference in the effect of eating schedule on postprandial digestive well-being with 80% power and 5% significance threshold.

In each group, means and standard errors of the measured variables were calculated. The Kolmogorov–Smirnov test was used to check the normality of the data distribution. Parametric normally distributed data were compared by Student’s *t*-test for paired or unpaired data; otherwise, the Wilcoxon signed rank test was used for paired data, and the Mann–Whitney *U* test was used for unpaired data.

Temporal responses to meal ingestion on each study day were analysed using one-way ANOVA for repeated measurements of the whole curve (10 minutes pre and 60 minutes postprandial period). ANOVA assumptions were checked, and, when sphericity was violated, Greenhouse–Geisser correction was applied [18]. Comparisons between groups (gender effect) and between afternoon and morning meals (schedule effect) were performed with a two-way repeated measures ANCOVA (dependent variable: postprandial sensations scores; within-subjects factors: time and meal schedule; between-subjects factor: gender; covariate: premeal scores). To test whether the effect of eating schedule was different in women and men, a gender by schedule interaction effect was included. Differences were considered significant at a *p* value <0.05 [18].

## 3. Results

### 3.1. Demographics and Study Conduction

Participants were in the 21–33 years age range without differences between women and men. Body weight and height were 60 ± 3 Kg and 169 ± 1 cm in women, and 71 ± 2 Kg and 175 ± 1 cm, respectively, in men. The body mass index range was 18.8–24.9 Kg/m^2^ in women and 21.6–24.1 Kg/m^2^ in men. All participants had a normal bowel habit and scored HAD, PAS, and DEBQ within the normal range. Each study day, participants confirmed compliance with the dietary instructions. In three participants (two women, one man), the tests were performed on consecutive days and the rest had at least a 1-day washout in between the tests. All participants completed the studies and were included for analysis.

### 3.2. Homeostatic and Hedonic Sensations in Response to Meal Ingestion

#### 3.2.1. Women: Within-Group Analysis

1. Meal Effect: Responses to the Afternoon Meal

**Figure 1:**
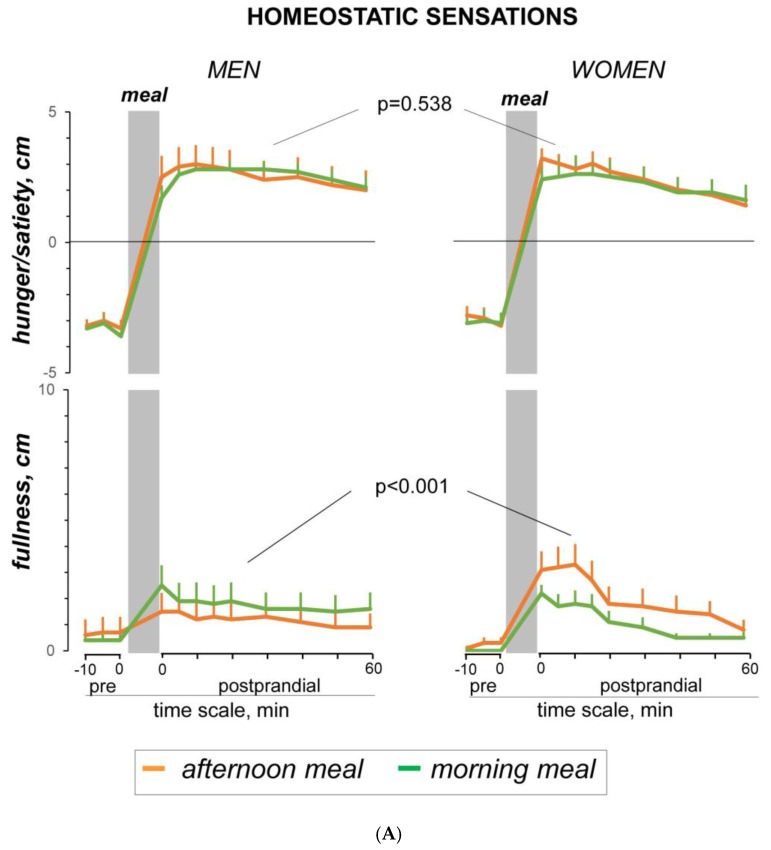

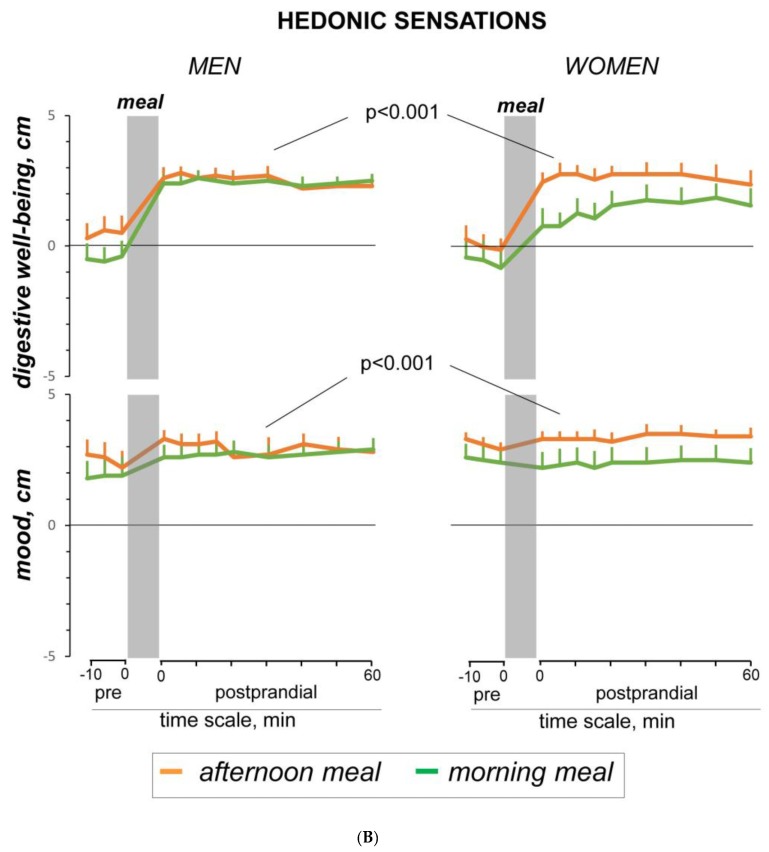
The effect of ingestion time on the postprandial experience. (**A**) Homeostatic sensations. (**B**) Hedonic sensations. Perceptions measured on 10-cm scales before (−10 to 0 minutes) and after (0 to 60 minutes) the meal; ingestion time 12 min. In women, the sensory experience induced by the probe meal, particularly postprandial satisfaction, was weaker when eaten at an unconventional time for breakfast. The effect of meal schedule was more pronounced in women than men. The main gender effect by repeated measures ANCOVA is shown. Dependent variable: postprandial scores. Covariate: pre-meal scores.

**Figure 2:**
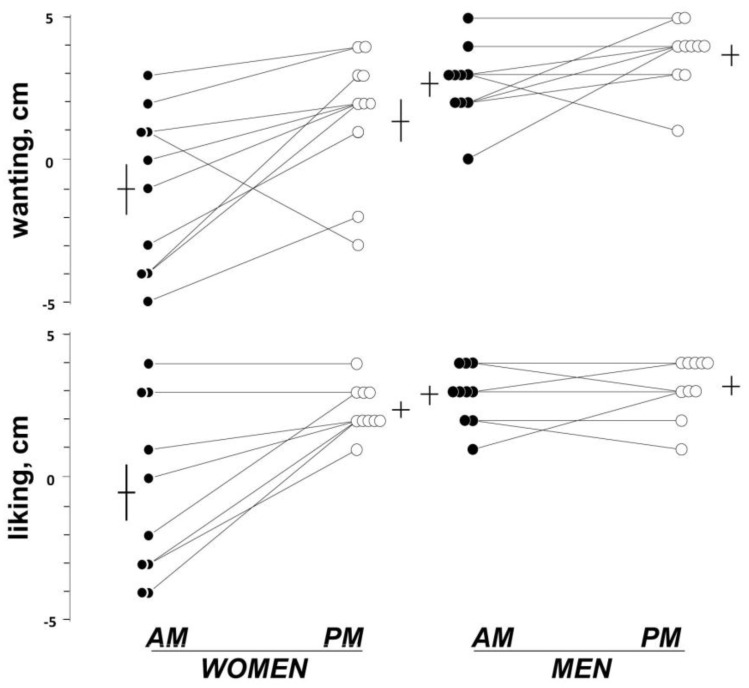
The effect of ingestion time on meal wanting and liking. Perceptions measured on 10-cm scales. By changing the ingestion schedule from the afternoon to the morning, meal liking decreased in women but not in men, and this gender difference was statistically significant (*p* = 0.008); similarly, the desire to eat also decreased in women but not in men, although the difference did not reach statistical significance (*p* = 0.109).

Before the afternoon meal (at the habitual eating time), women were hungry and in a positive mood (Figure 1). Before ingestion, they expressed desire to eat the meal and after ingestion they liked the meal (positive wanting and liking scores; Figure 2). Meal ingestion induced satiety, fullness, and a sensation of digestive well-being (time effect *p* ≤ 0.005 for all; Figure 1).

2. Schedule Effect: Differences between Afternoon and Morning Tests in Women

No significant differences in premeal sensations were observed between the afternoon and morning tests. However, wanting scores before the meal and liking scores after ingestion were significantly lower for the morning than for the afternoon meal (Figure 2). The effects of the morning meal on digestive well-being were significantly weaker as compared to the afternoon meal (main schedule effect F(1,44.9) = 8.78; *p* = 0.009). The morning meal tended to induce lower fullness scores than the afternoon meal, but the difference was not statistically significant (main schedule effect F(1,10.9) = 1.79; *p* = 0.198). The schedule effect on digestive well-being (difference in afternoon versus morning meals measured by the area under the curve) did not correlate with the schedule effect on meal liking *R* = −0.4; *p* = 0.280).

#### 3.2.2. Men: Within-Group Analysis

1. Meal Effect: Responses to the Afternoon Meal

Before the meal, men reported hunger and a positive mood. They reported positive meal wanting and liking (Figure 2). Meal ingestion induced significant satiety and digestive well-being (time effects; *p* ≤0.002 for both) without changes in fullness and mood scores (Figure 1).

2. Schedule Effect: Differences between Afternoon and Morning Tests in Men

Digestive well-being before the meal was somewhat lower in the morning than in the afternoon (−0.5 ± 0.6 score versus 0.5 ± 0.5 score, respectively; *p* = 0.015); no other differences in premeal sensations, meal liking, and wanting scores were detected between study days. In contrast to the afternoon meal, the morning meal induced a fullness sensation (main time effect F(1.9,17) = 3.69; *p* = 0.049), although the difference between tests did not reach statistical significance (main schedule effect F(1,19.7) = 4.12; *p* = 0.058). In fact, no significant differences in the postprandial sensations in response to the morning and the afternoon meals were detected.

#### 3.2.3. Gender Effect: Between-Group Analysis in Women versus Men

1. Meal Effect: Responses to the Afternoon Meal

In the afternoon test, no differences in premeal sensations, meal wanting, and meal liking were detected between women and men. Despite the fact that the meal induced significant fullness in women but not in men, no statistical gender differences in the postprandial sensations were detected.

2. Schedule Effect: Differences between Afternoon and Morning Tests

By changing the ingestion schedule from the afternoon to the morning, meal liking decreased in women but not in men, and this difference was statistically significant (*p* = 0.008); similarly, meal wanting also decreased in women but not in men, but the difference did not reach statistical significance (*p* = 0.109). The effect of the eating schedule (schedule effect) was significantly more pronounced in women for fullness (gender by schedule effect F(1,55) = 14.9; *p* <0.001), digestive well-being (F(1,36.8) = 22.3; *p* <0.001), and mood (F(1,12.4) = 13.8; *p* <0.001) as compared to men; the (lack of an) effect of eating schedule on satiety was similar in women and men (*p* = 0.538 in women versus men).

### 3.3. Additional Outcomes

Other sensations. Meal ingestion tended to increase somnolence and reduce anxiety scores without abdominal discomfort/pain. A gender by schedule effect was detected for anxiety (F(1,11.9) = 10.9; *p* = 0.001) but not for somnolence (*p* = 0.261).

Physiological responses. Overall (*n* = 20), ingestion of the afternoon meal reduced diastolic blood pressure (F(1.4,2.7) = 5.7; *p* = 0.016) and heart rate (F(2.7,51) = 35; *p* <0.001) and increased vagal tone (F(2.2,41) = 6.5; *p* = 0.003). The antral cross-sectional area increased immediately after meal ingestion and decreased during the postprandial period (F(2.1,43) = 83; *p* <0.001), while gallbladder volume decreased from premeal to 60 minutes postprandial measurements (by 59 ± 4%; *p* <0.001). No differences in the physiological responses induced by the afternoon and morning meals were detected (no schedule effects), and this lack of schedule effects applied equally to women and men (no gender effects).

## 4. Discussion

Our data indicate that women are more susceptible to changes in meal schedule than men. A meal concordant with the local lunch habits induced consistent homeostatic and hedonic responses when eaten on the habitual schedule in the afternoon. In women, the same meal induced weaker responses, particularly with less postprandial satisfaction, when eaten at an unconventional time for breakfast. In contrast to women, men were resilient to the schedule effect and experienced the same homeostatic and hedonic sensations regardless of the timing of ingestion. These gender differences (a schedule effect in women versus a lack of a schedule effect in men) were statistically significant, but the mechanisms involved are not clear and several factors may be considered. To note, the differences in the sensory experience were not associated with changes in physiological responses to the meal; conceivably, the homeostatic control of meal processing is less susceptible to external inferences.

In a previous study, we detected differences in the responses to a palatable meal in women and men [9]. A meal load that induced a pleasant fullness sensation in women was insufficient to achieve the same degree of homeostatic and hedonic reward in men. In contrast to these previous data, in the current study ingestion of the probe meal on the habitual schedule induced the same responses in women and in men. Hence, gender differences in the schedule effect were not related to differences in the response to the reference meal. To note, the meal load in the previous study (750 Kcal, 55 g fat) was heavier than the probe meal in the current study (550 Kcal, 25 g fat), suggesting that gender differences manifest under forced conditions, such as heavy meals (as in the previous study) or extemporaneous meals (as in the current study). Conceivably, a more offensive disruption of meal schedule would also affect the postprandial response in men.

Different responses to the same meal eaten at different times of the day may be related to the subject’s circadian clock. A network of biological pacemakers in the organism adapts metabolic and behavioural activity to external circadian variations, such as daily light cycles and rhythmic food intake [19,20]. Some data indicate that the metabolic response to a meal depends on the time of ingestion in relation to the individual’s circadian cycle [6,7,8]. Differences in the circadian clock in women and men have been described, and these gender-related chronotypes may explain the differential effects of meal schedule in women versus men [19,21,22].

It is not clear to what extent eating habits also influence the response to a meal depending on the ingestion time. Eating habits in our area feature a relatively light continental-type breakfast with a predominant sweet component and a heavier, savoury lunch, i.e., a hot meat and legume-based meal is not traditionally part of breakfast. The probe meal in our study was designed as a prototype lunch-meal according to local habits.

The predisposition and the responses to a meal not only depend on chronobiology and habits, but also cognitive factors may play an important role. In a previous study, we demonstrated that a cognitive-sensory intervention changed both the palatability and the postprandial response to a meal [5]. Indeed, an educative intervention enhanced both homeostatic and hedonic responses to a probe meal; an effect similar to the difference in women between the morning and afternoon meals observed in the current study. Educational and cognitive factors probably determine meal expectancy and acceptability at different daytimes [1].

The various effects of eating time observed in women (effects on meal wanting, meal liking, digestive well-being, fullness, and anxiety) could be inter-related. We have previously shown that meal palatability influences the postprandial response [4]: reduced liking was associated with less postprandial satisfaction (lower digestive well-being scores), a similar effect as with the less-likable and less-rewarding morning meal. However, in the previous study, lower palatability was associated with increased satiety and fullness, the opposite effect than with the morning meal. Hence, the schedule effect on meal liking does not account for the rest of the observed schedule effects.

Appetite has been shown to influence the responses to a meal: less hunger before the meal results in higher satiety/fullness and less satisfaction after ingestion [3]. Considering these data, the present study was designed to have the participants with similar hunger sensation before the morning meal (after a standard dinner the night before) and before the afternoon meal (after a standard breakfast 5 hours before). Furthermore, no differences in hunger sensation at the time of the probe meals were detected between women and men, and this is concordant with previous studies using the same breakfast [1].

We strived to control the factors that may influence the response under study, but we acknowledge that some of the tests were conducted on consecutive days with no washout, leaving open the possibility that the second test day was affected by the first. Furthermore, in this proof-of-concept study, the responses to a single probe meal were evaluated, while evaluation of the full extent of the schedule effect would require a more complex dose-response study with graded caloric loads.

A large body of evidence indicates that meal timing can affect homeostasis, including the sleep pattern, body temperature, alertness, visceral sensitivity, and energy balance [6,7,8,23,24]. In this regard, chrono nutrition is particularly relevant for the well-being of the organism and in the prevention of metabolic pathogenesis, such as obesity, diabetes, and metabolic syndromes. Several studies have shown the advantage of distributing the caloric intake at an earlier circadian time [7,8], and our study suggests that this approach may entrain specific strategies in women and men.

## Figures and Tables

**Table 1:** The probe meal.

	Total	Total	FAT	PROT	CHO
	g	kcal	g	g	g
stewed beans	300	460.2	24.4	22.8	29.9
bread	35	89	0.8	3.3	18
water	100	0	0.0	0.0	0
probe meal	435	549.2	25.2	26.1	47.9

PROT: proteins, CHO: carbohydrates.
